# Implicit attitudes toward death and associations with suicide risk in women with depression

**DOI:** 10.1192/j.eurpsy.2026.11439

**Published:** 2026-07-31

**Authors:** T. I. Medvedeva, S. N. Enikolopov, O. M. Boyko, O. Y. Vorontsova

**Affiliations:** clinical psychology, Federal State Budgetary Scientific Institution Mental health scientific center, Moscow, Russian Federation

## Abstract

**Introduction:**

The relevance of studying suicidal behaviour in patients suffering from endogenous mental illnesses is due to the high suicide rate in this group. The difficulties in assessing suicide risk are related to the fact that people who are going to commit suicide are often unable or unwilling to talk about it, which determines the need to use implicit methods.

**Objectives:**

The aim of the study was to investigate implicit preferences associated with death (categories ‘I’ + ‘Death’) in patients with depression with and without suicidal ideation.

**Methods:**

The study involved 132 female patients of the clinic with endogenous depression (age 16–23). We use modified “Test of Implicit Associations” (IAT), the IAT effect (referred to as Dscore) was calculated, positive IAT effect reflect a stronger associative connection between oneself and ‘death’ (Nock). All subjects were divided into two subgroups based on their IAT results: a subgroup without an IAT effect (N=105) and a subgroup with an IAT effect (N=27). The subgroups did not differ statistically in terms of age. Iowa Gambling Task (IGT), Expectancy-valence model was used for the result processing; Barratt Impulsiveness Scale (BIS-11); Body Investment Scale (BIS) - subscale ‘Protection’ was used to assess the tendency towards risky behaviour; Moral dilemmas (Greene); questions about suicidal intentions, thoughts of death, feelings of uncertainty about the future (rated on a Likert scale) were used.

**Results:**

Subgroups with and without an IAT effect showed differences in the severity of suicidal thoughts (2,2 in the group with a positive IAT effect compared to 1,1; p<0,000), thoughts about death and feelings of uncertainty about the future. The subgroup with a positive IAT effect also showed impaired ‘self-control’ and ‘planning’ (31,9±3,7 and 28,5±5,3 respectively p=0,02), which is confirmed by the data from the IGT (Expectancy-valence model parameter ‘learning’ 0,57±0,42 and 0,42±0,41 p=0,04, which shows memory of the past compared to the present, with higher values corresponding to rapid changes in behaviour, short associative memory, and rapid forgetting, which hinders long-term planning). The subgroup with an implicit ‘preference’ for death showed higher rates of risk-taking behaviour (BIS_ Protection). And in moral dilemmas, personal choices were significantly more often preferred in group with a positive IAT effect (5,2 compared to 3,7).

**Conclusions:**

It has been shown that the subgroup of subjects who implicitly associate themselves with ‘death’ are characterised by suicidal ideas, thoughts of death, and a sense of uncertainty about the future. This subgroup exhibits impaired self-control and planning, as well as a tendency towards risky behaviour. The implicit association of oneself with the death reduces the emotional connection to the death of another and makes it easier to make decisions that may harm another.

**Disclosure of Interest:**

None Declared

